# Supplementary material for: Optimized Lambda Exonuclease Digestion or Purification Using Streptavidin-Coated Beads: Which One Is Best for Successful DNA Aptamer Selection?
Source: Methods Protoc. 2022 Oct 29;5(6):89. doi: 10.3390/mps5060089 (PMC9680285; doi:10.3390/mps5060089)
Supplement: Supplementary file 1 [file mps-05-00089-s001.zip › mps-1830710-supplementary.pdf]

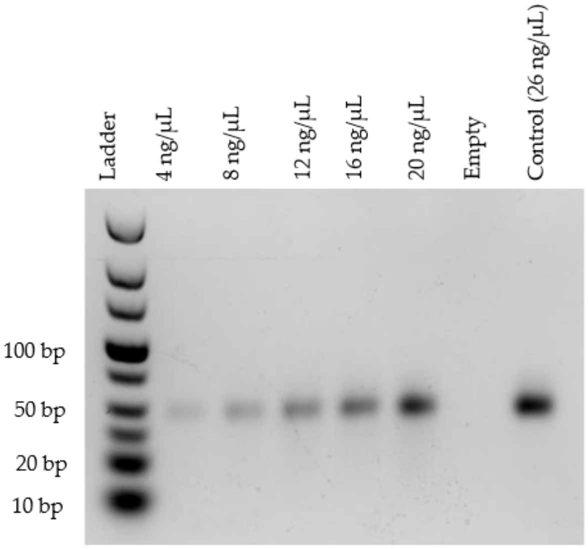

a	1731,9
b	-6005,9
**Concentration of control (ng/µL)**	26,07

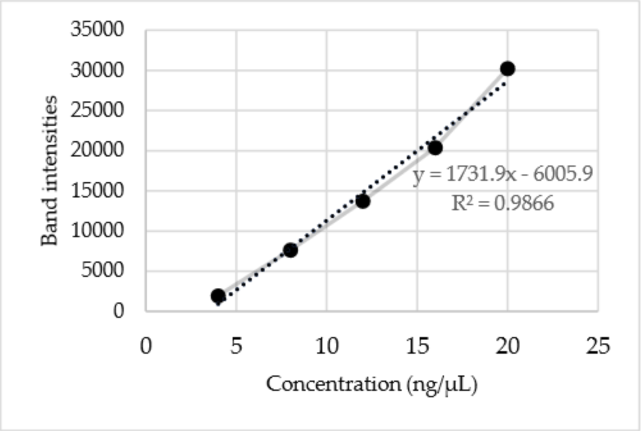

(a) **(b)**

Figure S1: Quantification of the control (library), using gel-based quantification. **(a):** Agarose gel analysis of the concentration range of the library. Products were revealed on a 4% agarose gel at 90 V for 2h (TAE 1X, GelRed®). **(b):** Confirmation of the control concentration using the correlation curve between band intensities (ImageJ software) and the concentration range.

Table S1: Average ssDNA concentration (ng/µL) obtained after purification using streptavidin-coated beads or lambda exonuclease digestion, before or after ethanol precipitation/ phenol-chloroform extraction.

	Purification of ssDNA using					
	Streptavidin-coated beads			Lambda exonuclease digestion		
**Quantification techniques**	**Before purification (n=10)**	**After ethanol precipitation (n=5)**	**After phenol chloroform extraction (n=5)**	**Before purification (n=10)**	**After ethanol precipitation (n=5)**	**After phenol chloroform extraction (n=5)**
Qubit	NA	3.7 ± 0.41	3.84 ± 0.35	NA	4.48 ± 0.56	5.16 ± 0.65
Nanodrop™	NA	9.14 ± 0.53	9.88 ± 0.78	NA	16.2 ± 0.43	17.5 ± 0.73
Gel-based quantification	8.8 ± 0.38	6.86 ± 0.62	7.94 ± 0.52	14.8 ± 0.29	11.6 ± 0.56	11.3 ± 0.75

Table S2: Average ssDNA recovery (%) obtained after purification using streptavidin-coated beads or lambda exonuclease digestion, before or after ethanol precipitation/ phenol-chloroform extraction.

	Purification of ssDNA using					
	Streptavidin-coated beads			Lambda exonuclease digestion		
**Quantification techniques**	**Before purification (n=10)**	**After ethanol precipitation (n=5)**	**After phenol chloroform extraction (n=5)**	**Before purification (n=10)**	**After ethanol precipitation (n=5)**	**After phenol chloroform extraction (n=5)**
Qubit	NA	18.5 ± 3.4	19.2 ± 2.0	NA	22.4 ± 2.5	25.8 ± 3.0
Nanodrop™	NA	45.7 ± 3.1	49.4 ± 5.7	NA	81.2 ± 7.8	87.5 ± 5.6
Gel-based quantification	44 ± 5.8	34.3 ± 6.4	39.7 ± 1.8	74 ± 9.3	58.1 ± 3.9	56.6 ± 6.0
Comparison of	Qubit/Nanodrop	< 0.001	< 0.001	Qubit/Nanodrop	< 0.001	< 0.001
quantification techniques	Qubit/Gel	< 0.001	< 0.001	Qubit/Gel	< 0.001	< 0.001
Student's *t*-test	Nanodrop/Gel	< 0.01	< 0.01	Nanodrop/Gel	< 0.01	< 0.01

*SD is expressed in %. The ssDNA quantification techniques were compared in a peer comparison by Student's statistical test. The differences in yields obtained between Qubit, Nanodrop™ and gel-based quantification were statistically significant, with values ranging from significant ($p<0.01$) to highly significant ($p<0.001$), depending on the techniques compared.
